# EBV induces persistent NF-κB activation and contributes to survival of EBV-positive neoplastic T- or NK-cells

**DOI:** 10.1371/journal.pone.0174136

**Published:** 2017-03-27

**Authors:** Honami Takada, Ken-Ichi Imadome, Haruna Shibayama, Mayumi Yoshimori, Ludan Wang, Yasunori Saitoh, Shin Uota, Shoji Yamaoka, Takatoshi Koyama, Norio Shimizu, Kouhei Yamamoto, Shigeyoshi Fujiwara, Osamu Miura, Ayako Arai

**Affiliations:** 1 Department of Hematology, Graduate School of Medical and Dental Sciences, Tokyo Medical and Dental University (TMDU), Bunkyo-ku, Tokyo, Japan; 2 Department of Laboratory Molecular Genetics of Hematology, Graduate School of Health Care Sciences, Tokyo Medical and Dental University (TMDU), Bunkyo-ku, Tokyo, Japan; 3 Division of Advanced Medicine for Virus Infections, National Research Institute for Child Health and Development, Setagaya-ku, Tokyo, Japan; 4 Department of Molecular Virology, Graduate School of Health Care Sciences, Tokyo Medical and Dental University (TMDU), Bunkyo-ku, Tokyo, Japan; 5 Virus Research Unit, Division of Medical Science, Medical Research Institute, Tokyo Medical and Dental University (TMDU), Bunkyo-ku, Tokyo, Japan; 6 Department of Comprehensive Pathology, Graduate School of Medical and Dental Sciences, Tokyo Medical and Dental University (TMDU), Tokyo, Japan; 7 Department of Allergy and Clinical Immunology, National Research Institute for Child Health and Development, Setagaya-ku, Tokyo, Japan; University of Nebraska-Lincoln, UNITED STATES

## Abstract

Epstein–Barr virus (EBV) has been detected in several T- and NK-cell neoplasms such as extranodal NK/T-cell lymphoma nasal type, aggressive NK-cell leukemia, EBV-positive peripheral T-cell lymphoma, systemic EBV-positive T-cell lymphoma of childhood, and chronic active EBV infection (CAEBV). However, how this virus contributes to lymphomagenesis in T or NK cells remains largely unknown. Here, we examined NF-κB activation in EBV-positive T or NK cell lines, SNT8, SNT15, SNT16, SNK6, and primary EBV-positive and clonally proliferating T/NK cells obtained from the peripheral blood of patients with CAEBV. Western blotting, electrophoretic mobility shift assays, and immunofluorescent staining revealed persistent NF-κB activation in EBV-infected cell lines and primary cells from patients. Furthermore, we investigated the role of EBV in infected T cells. We performed an *in vitro* infection assay using MOLT4 cells infected with EBV. The infection directly induced NF-κB activation, promoted survival, and inhibited etoposide-induced apoptosis in MOLT4 cells. The luciferase assay suggested that LMP1 mediated NF-κB activation in MOLT4 cells. IMD-0354, a specific inhibitor of NF-κB that suppresses NF-κB activation in cell lines, inhibited cell survival and induced apoptosis. These results indicate that EBV induces NF-κB-mediated survival signals in T and NK cells, and therefore, may contribute to the lymphomagenesis of these cells.

## Introduction

Epstein-Barr virus (EBV) is positive in some T- and NK-cell neoplasms, including extranodal NK/T-cell lymphoma nasal type (ENKL) [1], aggressive NK-cell leukemia (ANKL) [2], EBV-positive peripheral T-cell lymphoma, systemic EBV-positive T-cell lymphoma of childhood, and chronic active EBV infection (CAEBV) [3]. Systemic EBV-positive T-cell lymphoma of childhood and CAEBV were described for the first time as EBV-positive T-lymphoproliferative diseases (EBV-T-LPDs) of childhood in the WHO classification in 2008 [4]. In the classification revised in 2016, EBV-T-LPDs of childhood were divided into 2 disorders: systemic EBV-positive T-cell lymphoma of childhood, an aggressive one, and CAEBV, a more indolent one [3]. CAEBV is a disorder presenting persistent inflammation: fever, hepatitis, lymphadenitis, and vasculitis. CAEBV also harbors 2 characteristic skin symptoms: hypersensitivity to mosquito bites, and hydroa vacciniforme-like eruption [5]. The EBV-infected cells in CAEBV are clonally proliferating and under type 2 latency of the viral infection.

EBV is well known to infect B cells, thus promoting their survival and occasionally leading to B-cell neoplasm development. Therefore, EBV has been proposed to associate also with the development of EBV-positive T- or NK-cell neoplasms, although its role in disease development has not been elucidated. To clarify the role of EBV in the development of EBV-positive T- and NK-cell neoplasms, we focused on NF-κB. NF-κB is a dimeric transcription factor of the REL family members, RelA, RelB, c-Rel, p50, and p52 that mediates inflammatory and anti-apoptotic molecular signals [6, 7]. Once activated, NF-κB translocates to the nucleus, binds DNA, and regulates gene expression. Notably, NF-κB is constitutively activated in various types of cancer cells, including EBV-positive B-cell lymphoma cells and contributes to tumor development [8, 9]. Expression profiling and histochemical studies have reported that p50, a component of NF-κB, was located in the nucleus and may be potentially activated in the EBV-positive NK-cell neoplasm ENKL [10–13]. In EBV-positive B-cell lymphomas, EBV directly activates NF-κB via the viral protein LMP1 [8, 9]. As LMP1 is also expressed in EBV-positive T- and NK-cell neoplasms, we hypothesized that NF-κB is also constitutively activated by EBV in EBV-infected T- or NK-cells and thus would contribute to the development of the neoplasms.

In this study, we investigated the activation and roles of NF-κB in EBV-positive T or NK cells (EBV-T/NK-cells). We examined the direct effects of EBV on cell survival related NF-κB activity.

## Materials and methods

### Cells and reagents

The EBV-T/NK-cell lines SNT8, 15, and 16 and SNK6, established from EBV-T or NK-cell neoplasms, were maintained in Roswell Park Memorial Institute (RPMI) 1640 medium containing 10% fetal calf serum (10% FCS–RPMI) and human interleukin-2 (IL-2) as described previously [14]. MOLT4-DL cells harbor integrated forms of two lentivirus vectors: CS-κB-R2.2, which expresses the firefly luciferase gene in an NF-κB-dependent manner, and pCERp, which constitutively expresses the *renilla* luciferase gene under the control of the elongation factor-1α enhancer/promoter [15]. IL-2 was purchased from R&D systems (Abington, UK). The EBV-negative T cell lines Jurkat and MOLT4 were cultured in 10% FCS–RPMI, whereas the EBV-negative NK cell line KHYG1 was cultured in 10% FCS–RPMI supplemented with 175 U/ml of human recombinant IL-2. Phorbol 12-myristate 13-acetate (PMA) was purchased from Wako Pure Chemical Industries (Osaka, Japan). The specific inhibitor for NF-κB, IMD-0354 was purchased from Sigma-Aldrich (St. Louis, MO, USA)

### Diagnosis of CAEBV

CAEBV was diagnosed according to the following criteria: the presence of characteristic symptoms, an increased EBV DNA load in the peripheral blood (PB), and the detection of clonally proliferating EBV-positive T/NK-cells [5, 16]. Pathologically diagnosed ENKL,ANKL, and PTCL-NOS were excluded.

### Detection and isolation of EBV-infected cells from CAEBV patients

Detection and isolation of EBV-infected cells were performed as described previously [15]. Peripheral blood mononuclear cells (PBMCs) from patients with CAEBV were isolated by density gradient centrifugation using Separate-L (Muto Pure Chemical, Tokyo, Japan); the cells were then separated into CD4-, CD8-, and CD56-positive fractions using antibody-conjugated magnetic beads (IMag Human CD4, CD8, and CD56 Particles-DM; BD Biosciences, Sparks, MD, USA) according to the manufacturer’s instructions. The EBV DNA load in each fraction was then measured via real-time -polymerase chain reaction (PCR) [17] using a TaqMan system (Applied Biosystems, Foster City, CA, USA). We have described the marker used to isolate the infected cells from each patient in Table 1.

**Table 1:** Patients with EBV-positive T/NK-LPDs and analyses of EBV-infected cells in their peripheral blood.

Case	Gender	Age	Infected cell	Clinical findings	EBV DNA load of each fraction of PBMCs (copies/μgDNA)						The marker used for the isolation of the infected cells	The clonality of the infected cells
					total	CD4	CD8	CD56	CD14	CD19		
CD4-1	F	25	CD4	HMB	7.0 x 10^4^	2.2 x 10^5^	N.D.	N.D.	N.D.	N.D.	CD4	monoclonal
CD4-2	F	62	CD4	sCAEBV	3.2 x 10^4^	4.6 x 10^5^	N.D.	N.D.	N.D.	N.D.	CD4	monoclonal
CD8-1	F	21	CD8	sCAEBV	1.8 x 10^3^	N.D.	6.0 x 10^2^	N.D.	N.D.	N.D.	CD8	monoclonal
CD8-2	F	64	CD8	sCAEBV	2.6 x 10^5^	N.D.	1.2 x 10^6^	N.D.	N.D.	4.6 x 10^5^	CD8	monoclonal
CD8-3	F	64	CD8	sCAEBV	4.7 x 10^4^	N.D.	1.7 x 10^4^	N.D.	N.D.	7.6 x 10^3^	CD8	monoclonal
CD56-1	F	18	CD56	HMB	5.2 x 10^4^	N.D.	N.D.	1.1 x 10^6^	N.D.	7.5 x 10^4^	CD56	monoclonal
CD56-2	F	48	CD56	sCAEBV	8.6 x 10^4^	N.D.	N.D.	1.0 x 10^5^	N.D.	N.D.	CD56	monoclonal
CD56-3	M	24	CD56	sCAEBV	2.9 x 10^3^	N.D.	N.D.	2.1x 10^4^	N.D.	N.D.	CD56	monoclonal

M: Male, F: Female; EBV: Epstein-Barr virus, PBMCs: peripheral blood mononuclear cells; HMB: hypersensitivity to mosquito bites, sCAEBV: systemic chronic active Epstein-Barr virus infection; N.D.: not detected.

### Western blotting

Proteins were separated into cytoplasmic and nuclear fractions, as described below. After washing with phosphate buffered saline (PBS), cells were lysed in hypotonic buffer (50 mM Tris-HCl (pH 7.5), 5 mM EDTA, 10 mM NaCl, 1 mM NaF, 1 mM Na_3_VO_4_, and 0.05% NP-40). Lysates were centrifuged at 700 × *g* for 2 min, and the supernatants were collected as cytoplasmic protein fractions. Pellets were washed thrice with hypotonic buffer and lysed in SIP buffer (50 mM Tris-HCl (pH 7.5), 5 mM EDTA, 100 mM NaCl, 50 mM NaF, 1 mM Na_3_VO_4_, 40 mM β-glycerophosphate, and 1% Triton X-100). Lysates were centrifuged at 15000 rpm for 10 min, and supernatants were used as the nuclear protein fractions. Proteins were quantified using DC protein assay kit (Bio-Rad, Hercules, CA, USA) and equivalent protein quantities were subjected to western blotting. A p52 antibody was purchased from Upstate Biotechnology (Temecula, CA, USA). Antibodies against p50, RelA, RelB, c-Rel, α-tubulin, HSP-90, and YY1 were purchased from Santa Cruz Biotechnology (Santa Cruz, CA, USA).

### Immunofluorescence staining

Cells were fixed on slides via immersion in 4% paraformaldehyde for 10 min, followed by 3 washes in PBS and incubation with mouse monoclonal anti-LMP1, p52, p50, RelA, or RelB antibodies at room temperature. Next, slides were treated at room temperature with a Cy5-conjugated Affinipure donkey anti-mouse antibody (Jackson ImmunoResearch Laboratories, Inc., West Grove, PA, USA) to label anti-p52 and anti-RelA antibodies or a PE-conjugated goat anti-rabbit antibody (Southern Biotech, Birmingham, AL, USA) to label anti-p50 and anti-RelB antibodies. Nuclei were counterstained with ProLong Gold and DAPI (Invitrogen, Carlsbad, CA, USA).

### Image acquisition

Cytological analysis was performed using a confocal microscope (Fluoview FV10i-DOC, Olympus, Tokyo, Japan). Photographs were taken using a 60X objective lens (NA 1.35).

### Electrophoretic mobility shift assay (EMSA) and super-shift assay

EMSA was performed as described previously [18]. Briefly, nuclear extracts were incubated for 30 min at room temperature in binding buffer (10 mM N-2-hydroxyethylpiperazine-N'-2-ethanesulfonic acid (HEPES), 100 mM NaCl, 1 mM ethylenediaminetetraacetic acid (EDTA), 1 mM dichlorodiphenyltrichloroethane (DTT), 2.5% glycerol, 0.5 μg of poly dI-dC) mixed with 0.5 ng of a ^32^P-labeled κB factor (KBF-1) probe derived from the H-2Kb promoter26 probe. For super-shift assays, nuclear extracts were incubated with anti-p50, anti-p52, anti-RelA, anti-RelB antibodies, purified rabbit IgG and mouse IgG (Cedarlane Laboratories Ltd., Burlington, NC, USA), or anti-cRel antibody (N) (Santa Cruz Biotechnology) for 30 min on ice before incubation with the labeled κB factor probe. Samples were run on a polyacrylamide gel containing 2.5% glycerol in 0.5× Tris-borate-EDTA buffer (TBE).

### In vitro EBV infection assay

MOLT4 or MOLT4-DL cells were infected with EBV as described previously [19, 20]. Briefly, spontaneously produced EBV was prepared from the culture medium of B95-8 cells. Specifically, 3 × 10^7^ cells were incubated in 200 mL of 10% FCS RPMI for 7 days. The virus-containing supernatant was centrifuged at 10,000 *g* and 4°C for 1 h to precipitate the virus. The pellet was suspended in 1 mL of 0.5 mM phosphate buffer (pH 8.0), loaded on a discontinuous (5–30%) dextran gradient, and centrifuged at 35,000 × *g* and 4°C for 2 h. The 15% dextran fraction, which contained the virus, was removed with a syringe. One mL of 10% FCS–RPMI was added to the fraction, which was subsequently filtered (pore size: 0.45 μm). Recipient cells (2 × 10^6^ to 1 × 10^7^) were incubated in 1 or 5 mL of this suspension for 1 h, rinsed twice with culture medium (10% RPMI), and used in the assay. Infection was verified by EBV DNA quantification and immunofluorescence staining of EBV nuclear antigen1 (EBNA1) as described previously with a polyclonal FITC-conjugated rabbit anti-human C3c complement antibody (Dako, Glostrup, Denmark) [21].

### PCR assay for EBV protein-encoding mRNA

RT-PCR for the detection of mRNA encoding the viral proteins LMP1, LMP2A, LMP2B, and EBNA1 was performed according to previous reports [20, 22].

### Plasmids

Plasmids for EBV-encoded proteins were generated from the EBV-positive cell line B95-8 as described previously [23]. The reporter plasmid used to detect NF-κB activation, pNF-κB-Luc, was purchased from Stratagene (Santa Clara, CA, USA); the control *Renilla luciferase* plasmid pRL-SV40 was purchased from Promega (Madison, WI, USA).

### Transfection

MOLT4 cells were transfected with plasmids encoding the viral proteins by electroporation as described previously [23]. SNT8, SNT15, SNT16, and SNK6 cells were transfected with the reporter plasmids by nucleofector kit using T-023 and X-001 program (Lonza Japan, Tokyo, Japan).

### Luciferase reporter assay

Assays of transiently transfected cells were performed as described previously using Dual-Luciferase Reporter Assay System (Promega, Tokyo, Japan) according to the manufacturer’s instructions. [24].

### XTT assay

The XTT assay was performed according to the sodium 3V-[1-(phenylaminocarbonyl)-3,4-tetrazolium]-bis(4-methoxy-6-nitro)-benzene sulfonic acid hydrate (XTT) colorimetric method and employed the Cell Proliferation Kit II (Roche Molecular Biochemicals, Indianapolis, IN, USA) according to the manufacturer’s instructions.

### Viability detection assay

Cell viability was examined by trypan blue staining.

### Apoptosis detection assay

Apoptosis detection was performed using the TACS^®^ Annexin V-FITC apoptosis detection kit (Trevigen, Gaithersburg, MD, USA) according to the manufacturer’s instructions.

### Statistical analysis

For the statistical analyses, the Mann–Whitney test was performed using Kyplot 5.0 software (KyensLab Inc., Tokyo, Japan).

### Ethics statement

This study was approved by the ethical committee of Tokyo Medical and Dental University. The approval number was 213. Written informed consent was obtained from each patient.

## Results

### NF-κB activation in EBV-positive T and NK cell lines and EBV-positive T and NK cells obtained from patients

**Fig 1:**
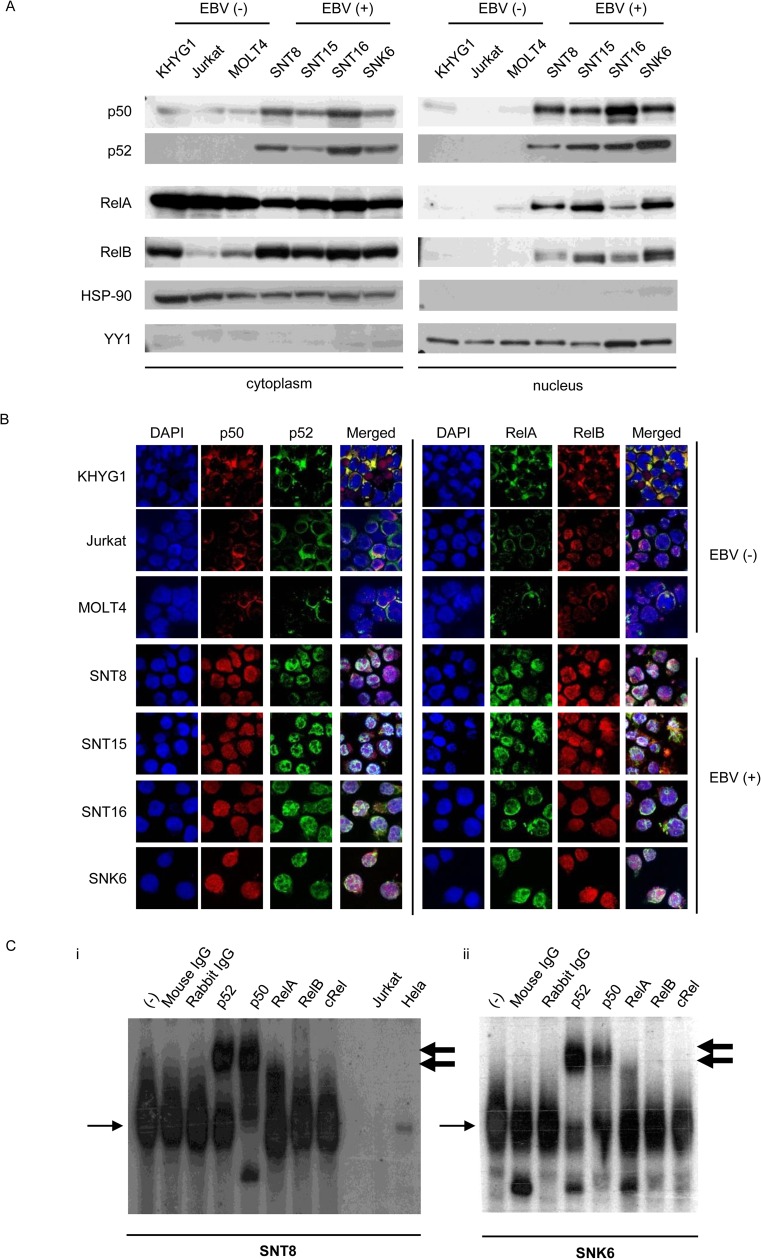
NF-κB activation in Epstein-Barr virus positive T or NK cells. (A) Western blotting for NF-κB protein localization in Epstein-Barr virus (EBV)-positive T or NK cell (EBV-T/NK cell) lines. The same samples were electrophoresed twice and transferred onto two membranes: one membrane for anti-p50 staining, and the other for p52, RelA, and RelB antibody staining. HSP90 and YY1 were proteins that localized to the cytoplasm and nucleus, respectively. (B) Immunofluorescent staining for NF-κB protein localization in EBV-T/NK cell lines. Immunofluorescent staining with anti-p50, p52, RelA, and RelB antibodies was performed as indicated. DAPI was used for nuclear staining. The EBV-negative cell lines, KHYG1, Jurkat, and MOLT4, were used as negative controls. Cells were analyzed via confocal microscopy. (C) Electrophoretic mobility shift assay of SNT8 (i) and SNK6 (ii) cells. Nuclear extracts were obtained, incubated with a KBF-1 probe containing the NF-κB binding sequence with or without indicated antibodies, and subjected to the assay. The thin, black arrows indicate KBF-1-binding NF-κB proteins. The thick, black arrows indicate supershifted bands. Nuclear extracts of Jurkat cells and HeLa cells treated with 10 ng/ml of TNF-α for 30 min were used as controls.

We investigated NF-κB activation in EBV-T/NK cell lines established from EBV-positive T- of NK-cell lymphoid neoplasms patients. First we performed the immunoblotting assay. In these cells, we found that p50, p52, RelA, and RelB existed in the nucleus under maintenance conditions, while KHYG1, Jurkat and MOLT4 cells, which were EBV-negative NK and T cell lines, did not show or showed a little nuclear localization of these molecules under these conditions (Fig 1A). We also investigated the intracellular localization of the proteins by immune-fluorescence staining. As shown in Fig 1B, p50, p52, RelA, and RelB were detected in the nucleus in the EBV-positive cell lines, whereas not or very weak in the EBV-negative cell lines. In addition, electrophoretic mobility shift assay (EMSA) using oligonucleotide probes encoding for the NF-κB-binding sequence revealed increased DNA-binding activity of nuclear NF-κB in SNT8 and SNK6 cells (Fig 1C, the leftmost lane in each panel). Supershifted bands demonstrated that NF-κB–DNA binding complexes in these cells involved p50, p52, RelA (4^th^ to 6the lanes in each panel). Thus, NF-κB was activated in these EBV-T/NK cell lines under maintenance conditions.

**Fig 2:**
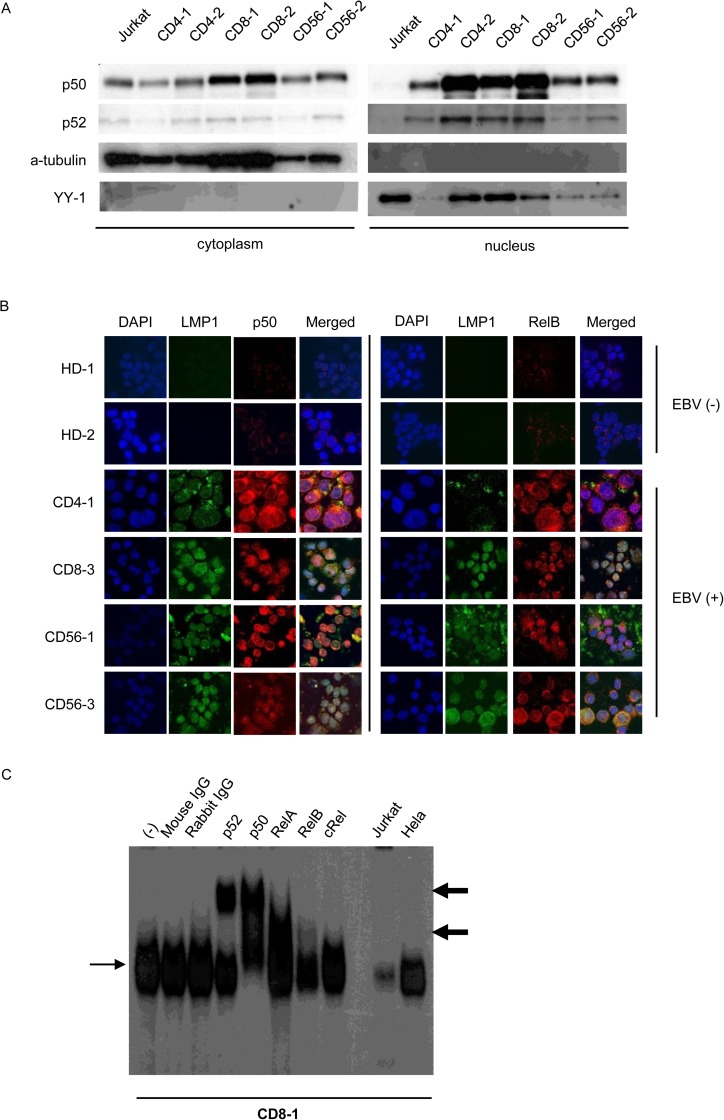
NF-κB activation in Epstein-Barr virus positive T or NK cells from patients with EBV-positive T or NK cell lymphoproliferative disorders. (A) Western blotting for p50 and p52 protein localization in EBV-positive T or NK cells purified from patient peripheral blood mononuclear cells (PBMCs) using antibody-conjugated magnetic beads and immediately used in the assay. (B) Immunofluorescent staining for NF-κB protein localization in patient PBMCs. EBV-positive T or NK cells purified from patient PBMCs using antibody-conjugated magnetic beads were immediately subjected to immunofluorescent staining with anti-LMP1 and anti-p50 or anti-LMP1 and RelB antibodies. DAPI was used for nuclear staining. PBMCs from healthy donors were used as negative controls. Cells were analyzed via confocal microscopy. (C) Electrophoretic mobility shift assay of PBMCs from CD8-1. Nuclear extracts were obtained, incubated with KBF-1 containing the NF-κB binding sequence with or without indicated antibodies, and subjected to the assay. Thin, black arrows indicate KBF-1-binding NF-κB proteins. Thick, black arrows indicate supershifted bands. Nuclear extracts of Jurkat cells and HeLa cells treated with 10 ng/ml of TNF-α for 30 min were used as controls.

Next we examined NF-κB activation in EBV-positive primary cells from CAEBV patients, whose EBV-positive T- or NK-cells were detected in the peripheral blood (PB). In this study, 8 CAEBV patients (aged 18–65 years; 1 males, 7 females; 5 T- and 3 NK-cell types; CD4 type: n = 2, CD8 type: n = 3, and CD56 type: n = 3) were investigated. The clinical findings, the phenotype of the infected cells, and the EBV DNA load of them were presented in Table 1. The flow cytometry of the peripheral blood lymphocytes from each patient was demonstrated in S1 Fig. Clonal proliferation of the infected cells was detected in all patients. EBV DNA load of PMBCs of the patients was 1.8 ×10^3^–2.6 ×10^5^ (mean 6.9×10^4^) copies/μg DNA. According to Kimua et al., the mean EBV DNA load of PMBCs of immunocompetent individuals was 10^1.2^ copies/μg DNA and that of CAEBV was 10^3.7^ copies/μg DNA [17]. Our cases had greater viral load than the cases characterized in this previous work. EBV DNA was detected not only in T-or NK-cell fraction but also in CD19-positive cell fraction in CD8-2, CD8-3, and CD56-1 patients. However, the LPD lesions of these patients contained mostly EBV-positive CD8- or CD56-positive cells (S2 Fig). Therefore, we concluded that these patients did not have B-cell neoplasms. The EBV positive T or NK cell fractions were purified from the peripheral blood of six patients by antibody-conjugated magnetic beads as described in Table 1, fractionated into nuclear and cytoplasmic fractions, and subjected to western blotting. As shown in Fig 2A, nuclear localization of p50 and p52 was detected in these samples. Immune fluorescence staining for the infected cells purified as described above showed the localization of p50, and RelB in the nucleus similar to that observed in the cell lines, whereas no nuclear localization of these proteins was seen in PBMCs from the healthy donors (Fig 2B). EMSA for the purified infected cells of case CD8-1 also demonstrated DNA binding activity of nuclear NF-κB. Supershifted bands demonstrated that NF-κB–DNA binding complexes involved p50, p52, RelA, and RelB. These results indicated that NF-κB was activated not only the cell lines, but also in the EBV-positive T- or NK-cells from CAEBV patients.

To confirm the latent infection, we investigated the expression of *BZLF1* gene that is expressed in the production infection of EBV, in PB of the patients. As shown in S3 Fig, we did not detect BZLF1 mRNA. The results indicated that high EBV DNA load in PB of the patients was not due to the viral production, but indicated the proliferation of EBV-infected T- or NK-cells.

### EBV infection induces NF-κB activation through LMP1 and suppresses serum depletion- or VP16-induced apoptosis in MOLT4 cells

**Fig 3:**
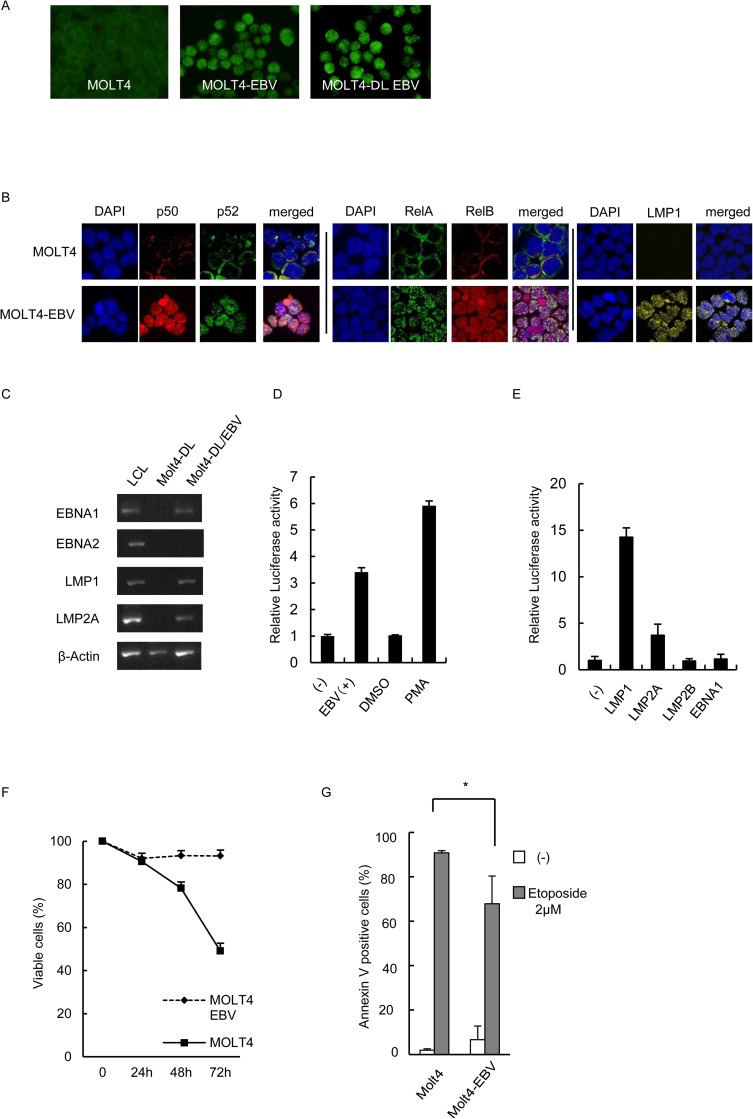
The effects of Epstein-Barr virus infection on MOLT4 cells (A) The original (left), Epstein-Barr virus (EBV)-infected MOLT4 (middle), and EBV-infected MOLT4-DL cells (right) were obtained for EBNA1 staining. Green fluorescent cells are EBNA1-positive. (B) Immunofluorescent staining for NF-κB protein localization in EBV-infected MOLT4 cells (MOLT4-EBV). The original and MOLT4-EBV cells were subjected to immunofluorescent staining with anti-p50, p52, RelA, and RelB antibodies as indicated. DAPI was used for nuclear staining. Cells were analyzed via confocal microscopy. (C) Reverse-transcriptase PCR analysis of EBV protein-encoding gene expression in EBV-infected MOLT4-DL cells. Infection was confirmed via detection of mRNAs encoding the viral proteins *EBNA1*, *LMP1*, *LMP2A*, and *LMP2B*. EBNA2 expression was not detected, and the infection type was considered latency type 2. (D) Dual luciferase assay for NF-κB in EBV-infected MOLT4-DL cells. As a positive control, MOLT4-DL cells were treated with 20 nM phorbol 12-myristate 13-acetate (PMA) for 18 h. (E) Luciferase reporter gene assay using expression vectors for EBV proteins in MOLT4 cells. MOLT4 cells were transfected with 10 μg of EBNA1, LMP1, LMP2A, LMP2B, or empty vector as indicated, along with 10 μg of pNF-κB-luc and 1 μg of pRLSV40. Twelve hours after transfection, cells were harvested for the dual luciferase assay. Luciferase activity was normalized to *Renilla luciferase* activity and expressed as an increase relative to the control. Data are shown as the means ± standard deviations of 3 independent experiments. (F) The original and EBV-infected MOLT4 cells were cultured in 10% FCS–RPMI or FCS-free RPMI. The time-dependent viable cell numbers were examined by trypan-blue staining. Data are shown as the means ± standard deviations of 3 independent experiments. (G) The original and EBV-infected MOLT4 cells were cultured for 24 h in 10% FCS–RPMI with or without 2 μM VP16. The viable and apoptotic cell numbers were then determined by Annexin V staining, and the percentage of Annexin V-positive cells was determined. * *p* = 0.03245. Each experiment was independently performed 3 times, and the average data are presented.

From these results, we hypothesized that EBV could directly induce NF-κB activation in T or NK cells, similar to that observed in B cells. To investigate this, we performed an *in vitro* EBV infection assay using the EBV-negative MOLT4 T cell line and the virus obtained from B95-8 cells. The average EBV-DNA copy number in the infected cells was 8.8 × 10^5^ copies/μg DNA. We used immunofluorescence staining to confirm the infection in MOLT4 cells according to EBNA1 (Fig 3A) expression. We used these infected cells for assays within 7 days. After infection, nuclear localization of P50, p52, RelA, and RelB was detected (Fig 3B). LMP1 expression was confirmed in the infected cells. We also examined NF-κB activity post-infection using a reporter assay. We performed an *in vitro* EBV infection assay with MOLT4-DL cells that express the firefly luciferase gene in an NF-κB -dependent manner and *renilla* luciferase gene under a constitutive promoter. In these cells, NF-κB activity can be monitored using a dual luciferase assay in which firefly luciferase activity can be normalized to *renilla* luciferase activity. Infection was performed and confirmed by EBV DNA detection via quantitative RT-PCR and EBNA1 protein detection in cells via immunofluorescence staining (Fig 3A). Results from these assays demonstrated that approximately 60% of the cells were EBV-positive. The average EBV DNA copy number in the EBV-infected MOLT4-DL cells was 1.6 × 10^5^ copies/μg DNA. As shown in Fig 3C, EBV-infected MOLT4-DL cells expressed mRNAs encoding the EBV proteins *LMP1*, *LMP2A*, *LMP2B*, and *EBNA1* and were considered latency type 2. We used these cells to investigate NF-κB-dependent transcriptional activity via dual luciferase assay. As shown in Fig 3D, NF-κB-dependent transcriptional activity increased significantly in the EBV-infected MOLT4-DL cells compared with that in uninfected cells, indicating that EBV infection induced NF-κB activation in MOLT4 cells. To investigate the molecular mechanism of this activation, we examined how the viral proteins LMP1, LMP2A, LMP2B, and EBNA1, expressed as latency type 2 in EBV-T/NK-cells, affected NF-κB. In MOLT4 cells, reporter assays involving expression vectors for these proteins revealed that LMP1, and to a lesser extent LMP2A, most significantly up-regulated NF-κB-dependent reporter gene expression, whereas the other viral proteins had no effect (Fig 3E). These results suggest that LMP1, and potentially LMP2A, is the main mediator of NF-κB activation in T cells.

Next, we examined the effects of EBV-infection on MOLT4 cell survival. We examined the time-dependent viability of the original and EBV-infected MOLT4 cells cultured in the serum free medium. As shown in Fig 3F, the viability of the original MOLT4 cells was decreased in time dependent manner, whereas that of EBV-infected MOLT4 cells was maintained. Next we performed an Annexin V apoptosis assay to detect etoposide-induced apoptosis in these cells. As shown in Fig 3G, the rate of apoptosis was significantly lower in the EBV-infected cells than in the original cells. Taken together, these results suggest that EBV induced NF-κB activation in T or NK cells to promote cell survival.

### IMD-0354 suppresses NF-κB activity and induces apoptosis in EBV-T/NK-cells

**Fig 4:**
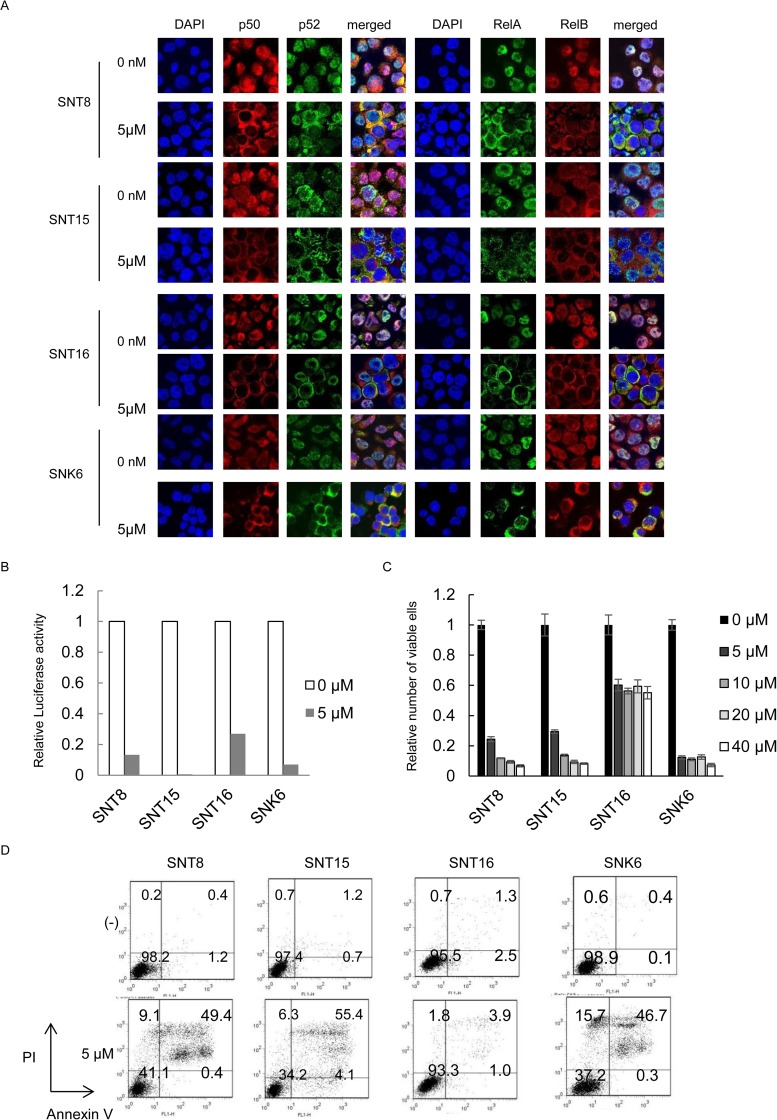
The effects of IMD-0354 on NF-κB activity and cell survival in Epstein-Barr virus positive T- or NK-cells. (A) Dual luciferase assay for NF-κB in SNT8, SNT15, SNT16, and SNK6 cells treated with IMD-0354. The cells were transfected with 10 μg of pNF-κB-luc and 1 μg of pRLSV40. Twelve hours after transfection, cells were treated with IMD-0354 for 24 hours and harvested for the dual luciferase assay. Luciferase activity was normalized to *Renilla luciferase* activity and expressed as an increase relative to the control. (B) SNT8, SNT15, SNT16, and SNK6 cells were treated with IMD-0354 for 24 hours and the viable cell numbers were estimated using the XTT assay and expressed in arbitrary units. Data are shown as the means ± standard deviations of 3 independent experiments. (C) SNT8, SNT15, SNT16, and SNK6 cells were treated with IMD-0354 as indicated for 24 h and subjected to immunofluorescent staining. NF-κB protein expression and localization were examined by immunofluorescent staining using anti-p50, p52, RelA, and RelB antibodies as indicated. DAPI was used for nuclear staining. Cells were analyzed via confocal microscopy. (D) SNT8, SNT15, SNT16, and SNK6 cells were treated with IMD-0354 as indicated for 24h and used in the assay. Cells were stained with Annexin V and PI and subsequently analyzed by flow cytometry.

To explore the contribution of NF-κB to EBV-infected cell survival, we used the specific inhibitor for NF-κB, IMD-0354 [25]. We investigated the effects of IMD-0354 on NF-κB activation in the EBV-positive cell lines. Luciferase reporter assays demonstrated the treatment suppressed NF-kB activation in the cells (Fig 4B). As shown in Fig 4C, the XTT assay revealed that the treatment reduced the number of living cells in the EBV-positive cell lines in a dose-dependent manner. Immunofluorescence staining also demonstrated that treatment with IMD-0354 inhibited the nuclear translocation of p50, p52, RelA, and RelB in EBV-positive cell lines (Fig 4A). The Annexin V assay showed that treatment with IMD-0354 remarkably induced apoptosis in the EBV-positive cell lines (Fig 4D). These results indicate that IMD-0354 suppressed NF-κB activity and induced apoptosis in EBV-T/NK cell lines.

## Discussion

The role of EBV in the lymphomagenesis of T and NK cells has remained undermined. The present study demonstrated a direct contribution of EBV infection to NF-κB activation in these cells. IMD-0354, an NF-κB inhibitor, suppressed the survival of EBV-positive T/NK cells in a dose-dependent manner. These results indicate that EBV may contribute to the lymphomagenesis of these cells through NF-κB activation.

We demonstrated the upregulation of NF-κB activation following LMP1 expression in a T-cell line. The mechanism of EBV-induced NF-κB activation has been mainly studied in LCLs. LMP1 is a viral membrane protein that resembles the proteins in the TNF receptor superfamily and induces NF-κB activation through interactions with the TNF receptor-associated factor (TRAF) and TNFR1-associated death domain protein (TRADD), which are associated with the TES1 and TES2 domains of LMP1, respectively [26, 27]. TRAF and TRADD induce NF-κB activation through NF-κB-inducing kinase and IκB kinases in the canonical and non-canonical pathways [26, 27]. Whether the same pathways contribute to EBV-induced NF-κB activation in T/NK cells remains unknown. Further evaluations such as a LMP1 knockdown assay are necessary for confirming the actual roles of this protein in CAEBV cells.

Some molecules whose expression is upregulated in EBV-infected cells may play indirect roles in NF-κB activation. SNK6 cells reportedly produce TNF-α [28], which activates NF-κB in T and NK cells. The serum TNF-α concentration was found to be significantly higher in patients with CAEBV than in healthy donors [29]. Secreted TNF-α can stimulate EBV-T/NK cells in an autocrine manner, thus promoting their survival, and may play a role in disease development, particularly in EBV-positive T- or NK-cell neoplasms. Furthermore, roles of the costimulatory molecules CD40 and CD137 should be examined. We previously reported that EBV infection induced ectopic but endogenous CD40 expression n T and NK cells thereby promoting their survival in an autocrine manner via a CD40 ligand, CD154, which was initially expressed on the surface of EBV-T/NK cells [19, 30]. In addition, we reported that EBV infection induced CD137 expression on the surface of T cells, which subsequently mediated anti-apoptotic signals [20]. Because NF-κB can be activated downstream of CD40 and CD137 [31, 32], EBV-induced CD40 or CD137 expression may play a role in NF-κB activation in EBV-T/NK cells.

Our present results indicate that EBV infection protects the infected cells from apoptosis and can render them immortal; however, EBV infection did not upregulate the proliferation of the infected cells (data not shown). This suggests that additional factors are necessary for the EBV-induced transformation of T or NK cells. Nakamura et al. reported that activation-induced cytidine deaminase (AID) expression was upregulated in PBMCs from patients with CAEBV. AID acts as a DNA and RNA editor and is essential for the somatic hypermutation and class-switch recombination of immunoglobulin genes. Dysregulated AID expression induces genomic mutation, leading to the development of B cell lymphoma. Interestingly, NF-κB can induce AID expression in B cells. The activation of NF-κB in T or NK cells following EBV infection may lead to the upregulation of AID. Ohshima et al. suggested a model for EBV-positive T- or NK-cell neoplasm development in which the malignant potential and subsequent lymphoma development increase gradually [33]. AID may contribute to EBV-induced tumor development in T or NK cells by cooperating with NF-κB-induced survival promoting pathways.

## Conclusions

EBV promotes cell survival and can induce neoplasm development via NF-κB activation not only in B cells but also in T and NK cells. The prognosis of EBV-positive T or NK-cell neoplasms remains poor, and further study is needed to clarify the mechanism of disease development and to apply these findings clinically.

## Supporting information

S1 Fig Flow cytometry for PBMCs from EBV-positive T- or NK-cell lymphoproliferative disorders patients. (TIF)

S2 Fig Histopathological specimen from the patients whose CD19-positive fraction in the peripheral blood was positive for EBV. (A-D) The lymph node of CD8-1. (A) Hematoxylin and eosin staining showed the infiltration of lymphocytes. (B) Immunochemical staining with anti-CD8 antibody (brown) showed that the infiltrating lymphocytes were positive for CD8. (C) *In situ* hybridization of Epstein–Barr virus-encoded mRNA (EBER) (brown). Infiltration of EBV-positive cells was detected. (D) Immunochemical staining with anti-CD20 antibody (brown). In comparison with CD8- and EBER-positive cells, CD20-positive infiltrating cells were markedly small in number. (original magnification, × 400).
(E-H) The skin lesion of CD8-2. (E) Hematoxylin and eosin staining showed the infiltration of lymphocytes. (F) Immunochemical staining with anti-CD8 antibody (brown) showed that the infiltrating lymphocytes were positive for CD8. (G) *In situ* hybridization of EBER (brown). Infiltration of EBV-positive cells was detected. (H) Immunochemical staining with anti-CD20 antibody (brown). In comparison with CD8- and EBER-positive cells, CD20-positive infiltrating cells were markedly small in number. (original magnification, × 400).
(I-L) The skin lesion of CD56-1. (I) Hematoxylin and eosin staining showed the infiltration of lymphocytes. (J) Immunochemical staining with anti-CD56 antibody (brown) showed that the infiltrating lymphocytes were positive for CD56. (K) *In situ* hybridization of EBER (brown). Infiltration of EBV-positive cells was detected. (L) Immunochemical staining with anti-CD20 antibody (brown). In comparison with CD56- and EBER-positive cells, CD20-positive infiltrating cells were markedly small in number. (original magnification, × 400).
(TIF)

S3 Fig Reverse-transcriptase PCR analysis of *BZLF1* gene expression in CAEBV patients. B95-8 cell and Jurkat cell were positive and negative control, respectively.
(TIF)

## References

[1] Chan JKC, Quintanilla-Martinez L, Ferry JA, Peh S-C. Extranodal NK/T-cell lymphoma, nasal type In: Jaffe E, Harris N, Stein H, editors. WHO Classification of Tumors of Haematopoietic and Lymphoid Tissues. Lyon IARC Press; 2008 285–288.

[2] Chan JKC, Jaffe ES, Ralfkiaer E, Ko Y-H. Aggressive NK-cell leukemia In: In: Jaffe E, Harris N, Stein H, editors. WHO Classification of Tumors of Haematopoietic and Lymphoid Tissues. Lyon. France: IARC Pre; 2008 276–277.

[3] Swerdlow SH, Campo E, Pileri SA, Harris NL, Stein H, Siebert R, et al The 2016 revision of the World Health Organization classification of lymphoid neoplasms. Blood. 2016;127(20):2375–90. PubMed Central PMCID: PMCPMC4874220. 10.1182/blood-2016-01-643569 26980727 PMC4874220

[4] Quintanilla-Martinez L, Kimura H, Jaffe ES. EBV-positive T-cell lymphoproliferative disorders of childhood In: Jaffe E, Harris N, Stein H, editors. WHO Classification of Tumors of Haematopoietic and Lymphoid Tissues. Lyon IARC Press; 2008 278–280.

[5] Kimura H, Ito Y, Kawabe S, Gotoh K, Takahashi Y, Kojima S, et al EBV-associated T/NK-cell lymphoproliferative diseases in nonimmunocompromised hosts: prospective analysis of 108 cases. Blood. 2012;119(3):673–86. 10.1182/blood-2011-10-381921 22096243

[6] Karin M, Cao Y, Greten FR, Li ZW. NF-kappaB in cancer: from innocent bystander to major culprit. Nat Rev Cancer. 2002;2(4):301–10. 10.1038/nrc780 12001991

[7] Baldwin AS. Series introduction: the transcription factor NF-kappaB and human disease. J Clin Invest. 2001;107(1):3–6. PubMed Central PMCID: PMCPMC198555. 10.1172/JCI11891 11134170 PMC198555

[8] Cahir-McFarland ED, Carter K, Rosenwald A, Giltnane JM, Henrickson SE, Staudt LM, et al Role of NF-kappa B in cell survival and transcription of latent membrane protein 1-expressing or Epstein-Barr virus latency III-infected cells. J Virol. 2004;78(8):4108–19. PubMed Central PMCID: PMCPMC374271. 10.1128/JVI.78.8.4108-4119.2004 15047827 PMC374271

[9] Keller SA, Hernandez-Hopkins D, Vider J, Ponomarev V, Hyjek E, Schattner EJ, et al NF-kappaB is essential for the progression of KSHV- and EBV-infected lymphomas in vivo. Blood. 2006;107(8):3295–302. PubMed Central PMCID: PMCPMC1432097. 10.1182/blood-2005-07-2730 16380446 PMC1432097

[10] Huang Y, de Reyniès A, de Leval L, Ghazi B, Martin-Garcia N, Travert M, et al Gene expression profiling identifies emerging oncogenic pathways operating in extranodal NK/T-cell lymphoma, nasal type. Blood. 2010;115(6):1226–37. PubMed Central PMCID: PMCPMC2826234. 10.1182/blood-2009-05-221275 19965620 PMC2826234

[11] Ng SB, Selvarajan V, Huang G, Zhou J, Feldman AL, Law M, et al Activated oncogenic pathways and therapeutic targets in extranodal nasal-type NK/T cell lymphoma revealed by gene expression profiling. J Pathol. 2011;223(4):496–510. 10.1002/path.2823 21294123

[12] Chan KK, Shen L, Au WY, Yuen HF, Wong KY, Guo T, et al Interleukin-2 induces NF-kappaB activation through BCL10 and affects its subcellular localization in natural killer lymphoma cells. J Pathol. 2010;221(2):164–74. 10.1002/path.2699 20235165

[13] Kanemitsu N, Isobe Y, Masuda A, Momose S, Higashi M, Tamaru J, et al Expression of Epstein-Barr virus-encoded proteins in extranodal NK/T-cell Lymphoma, nasal type (ENKL): differences in biologic and clinical behaviors of LMP1-positive and -negative ENKL. Clin Cancer Res. 2012;18(8):2164–72. 10.1158/1078-0432.CCR-11-2395 22371452

[14] Zhang Y, Nagata H, Ikeuchi T, Mukai H, Oyoshi MK, Demachi A, et al Common cytological and cytogenetic features of Epstein-Barr virus (EBV)-positive natural killer (NK) cells and cell lines derived from patients with nasal T/NK-cell lymphomas, chronic active EBV infection and hydroa vacciniforme-like eruptions. Br J Haematol. 2003;121(5):805–14. 12780797 10.1046/j.1365-2141.2003.04359.x

[15] Uota S, Zahidunnabi Dewan M, Saitoh Y, Muto S, Itai A, Utsunomiya A, et al An IκB kinase 2 inhibitor IMD-0354 suppresses the survival of adult T-cell leukemia cells. Cancer Sci. 2012;103(1):100–6. 10.1111/j.1349-7006.2011.02110.x 21951590 PMC11164137

[16] Okano M, Kawa K, Kimura H, Yachie A, Wakiguchi H, Maeda A, et al Proposed guidelines for diagnosing chronic active Epstein-Barr virus infection. Am J Hematol. 2005;80(1):64–9. 10.1002/ajh.20398 16138335

[17] Kimura H, Morita M, Yabuta Y, Kuzushima K, Kato K, Kojima S, et al Quantitative analysis of Epstein-Barr virus load by using a real-time PCR assay. J Clin Microbiol. 1999;37(1):132–6. PubMed Central PMCID: PMCPMC84187. 9854077 10.1128/jcm.37.1.132-136.1999 PMC84187

[18] Saitoh Y, Yamamoto N, Dewan MZ, Sugimoto H, Martinez Bruyn VJ, Iwasaki Y, et al Overexpressed NF-kappaB-inducing kinase contributes to the tumorigenesis of adult T-cell leukemia and Hodgkin Reed-Sternberg cells. Blood. 2008;111(10):5118–29. 10.1182/blood-2007-09-110635 18305221

[19] Imadome K, Shirakata M, Shimizu N, Nonoyama S, Yamanashi Y. CD40 ligand is a critical effector of Epstein-Barr virus in host cell survival and transformation. Proc Natl Acad Sci U S A. 2003;100(13):7836–40. PubMed Central PMCID: PMCPMC164674. 10.1073/pnas.1231363100 12805559 PMC164674

[20] Yoshimori M, Imadome KI, Komatsu H, Wang L, Saitoh Y, Yamaoka S, et al CD137 Expression Is Induced by Epstein-Barr Virus Infection through LMP1 in T or NK Cells and Mediates Survival Promoting Signals. PLoS One. 2014;9(11):e112564 10.1371/journal.pone.0112564 25409517 PMC4237363

[21] Reedman BM, Klein G. Cellular localization of an Epstein-Barr virus (EBV)-associated complement-fixing antigen in producer and non-producer lymphoblastoid cell lines. Int J Cancer. 1973;11(3):499–520. 4133943 10.1002/ijc.2910110302

[22] Imadome K, Yajima M, Arai A, Nakazawa A, Kawano F, Ichikawa S, et al Novel Mouse Xenograft Models Reveal a Critical Role of CD4 T Cells in the Proliferation of EBV-Infected T and NK Cells. PLoS Pathog. 2011;7(10):e1002326 PubMed Central PMCID: PMCPMC3197618. 10.1371/journal.ppat.1002326 22028658 PMC3197618

[23] Shirakata M, Imadome KI, Okazaki K, Hirai K. Activation of TRAF5 and TRAF6 signal cascades negatively regulates the latent replication origin of Epstein-Barr virus through p38 mitogen-activated protein kinase. J Virol. 2001;75(11):5059–68. PubMed Central PMCID: PMCPMC114910. 10.1128/JVI.75.11.5059-5068.2001 11333886 PMC114910

[24] Nosaka Y, Arai A, Miyasaka N, Miura O. CrkL mediates Ras-dependent activation of the Raf/ERK pathway through the guanine nucleotide exchange factor C3G in hematopoietic cells stimulated with erythropoietin or interleukin-3. J Biol Chem. 1999;274(42):30154–62. 10514505 10.1074/jbc.274.42.30154

[25] Tanaka A, Konno M, Muto S, Kambe N, Morii E, Nakahata T, et al A novel NF-kappaB inhibitor, IMD-0354, suppresses neoplastic proliferation of human mast cells with constitutively activated c-kit receptors. Blood. 2005;105(6):2324–31. 10.1182/blood-2004-08-3247 15561889

[26] Kieser A. Pursuing different 'TRADDes': TRADD signaling induced by TNF-receptor 1 and the Epstein-Barr virus oncoprotein LMP1. Biol Chem. 2008;389(10):1261–71. 10.1515/BC.2008.144 18713013

[27] Luftig M, Yasui T, Soni V, Kang MS, Jacobson N, Cahir-McFarland E, et al Epstein-Barr virus latent infection membrane protein 1 TRAF-binding site induces NIK/IKK alpha-dependent noncanonical NF-kappaB activation. Proc Natl Acad Sci U S A. 2004;101(1):141–6. PubMed Central PMCID: PMCPMC314152. 10.1073/pnas.2237183100 14691250 PMC314152

[28] Kanno H, Watabe D, Shimizu N, Sawai T. Adhesion of Epstein-Barr virus-positive natural killer cell lines to cultured endothelial cells stimulated with inflammatory cytokines. Clin Exp Immunol. 2008;151(3):519–27. PubMed Central PMCID: PMCPMC2276960. 10.1111/j.1365-2249.2007.03584.x 18190605 PMC2276960

[29] Arai A, Nogami A, Imadome KI, Kurata M, Murakami N, Fujiwara S, et al Sequential monitoring of serum IL-6, TNF-α, and IFN-γ levels in a CAEBV patient treated by plasma exchange and immunochemotherapy. Int J Hematol. 2012. 10.1007/s12185-012-1170-2 22983646

[30] Imadome K, Shimizu N, Arai A, Miura O, Watanabe K, Nakamura H, et al Coexpression of CD40 and CD40 ligand in Epstein-Barr virus-infected T and NK cells and their role in cell survival. J Infect Dis. 2005;192(8):1340–8. 10.1086/466530 16170750

[31] Rothe M, Sarma V, Dixit VM, Goeddel DV. TRAF2-mediated activation of NF-kappa B by TNF receptor 2 and CD40. Science. 1995;269(5229):1424–7. 7544915 10.1126/science.7544915

[32] Croft M. The role of TNF superfamily members in T-cell function and diseases. Nat Rev Immunol. 2009;9(4):271–85. PubMed Central PMCID: PMCPMC2737409. 10.1038/nri2526 19319144 PMC2737409

[33] Ohshima K, Kimura H, Yoshino T, Kim CW, Ko YH, Lee SS, et al Proposed categorization of pathological states of EBV-associated T/natural killer-cell lymphoproliferative disorder (LPD) in children and young adults: overlap with chronic active EBV infection and infantile fulminant EBV T-LPD. Pathol Int. 2008;58(4):209–17. 10.1111/j.1440-1827.2008.02213.x 18324913

