# Correction: EBV induces persistent NF-κB activation and contributes to survival of EBV-positive neoplastic T- or NK-cells

**DOI:** 10.1371/journal.pone.0182682

**Published:** 2017-08-01

**Authors:** Honami Takada, Ken-Ichi Imadome, Haruna Shibayama, Mayumi Yoshimori, Ludan Wang, Yasunori Saitoh, Shin Uota, Shoji Yamaoka, Takatoshi Koyama, Norio Shimizu, Kouhei Yamamoto, Shigeyoshi Fujiwara, Osamu Miura, Ayako Arai

There is an error in affiliation 4 for Yasunori Saitoh, Shin Uota, and Shoji Yamaoka. Affiliation 4 should be: Department of Molecular Virology, Graduate School of Medical and Dental Sciences, Tokyo Medical and Dental University (TMDU), Bunkyo-ku, Tokyo, Japan.
