# Supplementary figures and images for: EBV induces persistent NF-κB activation and contributes to survival of EBV-positive neoplastic T- or NK-cells
Source: PLoS One. 2017 Mar 27;12(3):e0174136. doi: 10.1371/journal.pone.0174136 (PMC5367708; doi:10.1371/journal.pone.0174136)

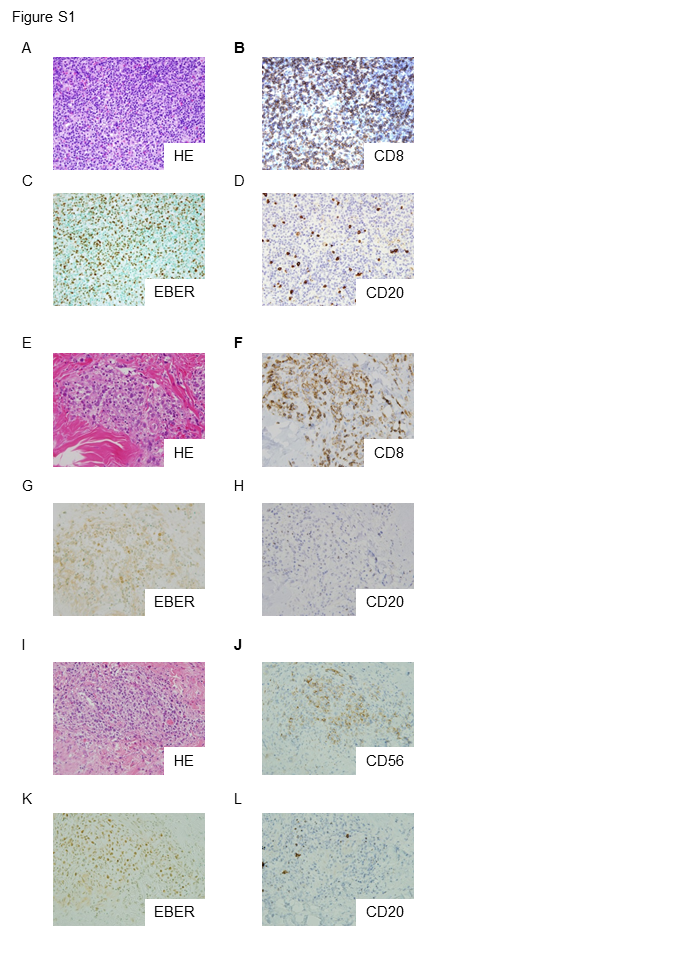

Supplement: S1 Fig — (TIF) [file pone.0174136.s001.TIF]

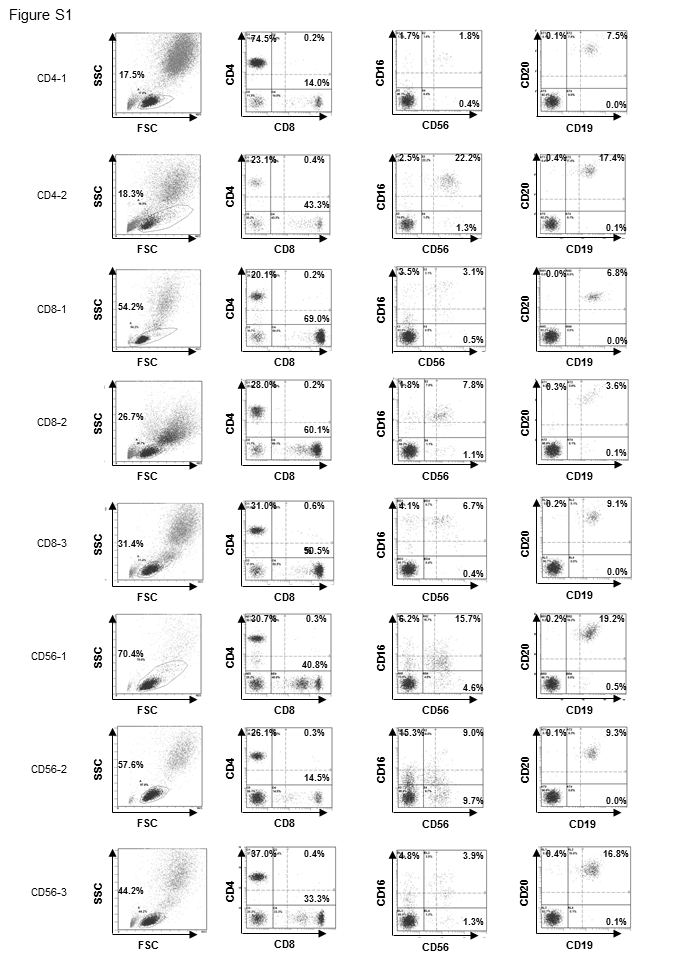

Supplement: S2 Fig — (A-D) The lymph node of CD8-1. (A) Hematoxylin and eosin staining showed the infiltration of lymphocytes. (B) Immunochemical staining with anti-CD8 antibody (brown) showed that the infiltrating lymphocytes were positive for CD8. (C) In situ hybridization of Epstein–Barr virus-encoded mRNA (EBER) (brown). Infiltration of EBV-positive cells was detected. (D) Immunochemical staining with anti-CD20 antibody (brown). In comparison with CD8- and EBER-positive cells, CD20-positive infiltrating cells were markedly small in number. (original magnification, × 400). (E-H) The skin lesion of CD8-2. (E) Hematoxylin and eosin staining showed the infiltration of lymphocytes. (F) Immunochemical staining with anti-CD8 antibody (brown) showed that the infiltrating lymphocytes were positive for CD8. (G) In situ hybridization of EBER (brown). Infiltration of EBV-positive cells was detected. (H) Immunochemical staining with anti-CD20 antibody (brown). In comparison with CD8- and EBER-positive cells, CD20-positive infiltrating cells were markedly small in number. (original magnification, × 400). (I-L) The skin lesion of CD56-1. (I) Hematoxylin and eosin staining showed the infiltration of lymphocytes. (J) Immunochemical staining with anti-CD56 antibody (brown) showed that the infiltrating lymphocytes were positive for CD56. (K) In situ hybridization of EBER (brown). Infiltration of EBV-positive cells was detected. (L) Immunochemical staining with anti-CD20 antibody (brown). In comparison with CD56- and EBER-positive cells, CD20-positive infiltrating cells were markedly small in number. (original magnification, × 400). (TIF) [file pone.0174136.s002.TIF]

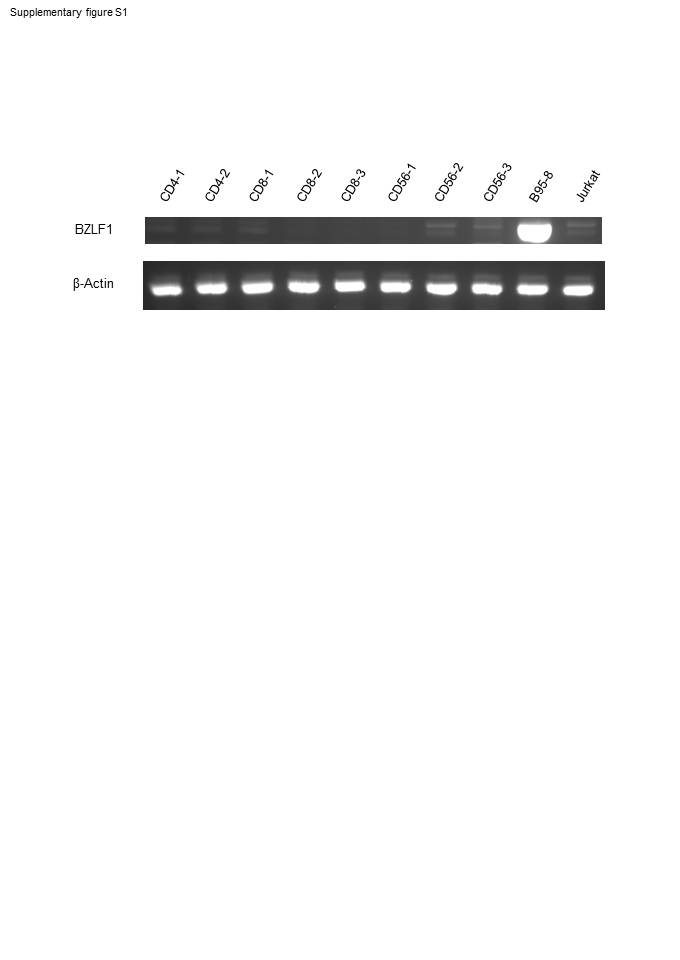

Supplement: S3 Fig — B95-8 cell and Jurkat cell were positive and negative control, respectively. (TIF) [file pone.0174136.s003.TIF]
